# Current Medical Diagnosis and Management of Vesicoureteral Reflux in Children

**DOI:** 10.5812/numonthly.13534

**Published:** 2013-11-05

**Authors:** Orcun Celik, Tumay Ipekci, Ozgu Aydogdu, Selcuk Yucel

**Affiliations:** 1 Urology Clinic, Kemalpasa State Hospital, Izmir, Turkey; 2 Department of Urology, Akdeniz University Medical School, Antalya, Turkey; 3 Department of Urology, Faculty of Medicine, Izmir University, Izmir, Turkey

**Keywords:** Vesico-Ureteral Reflux, Pyelonephritis, Cicatrix

## Abstract

Vesico-ureteral reflux (VUR) is presented in approximately %1 of children and is associated with an increased risk of pyelonephritis and renal scarring. Despite its prevalence and morbidity, many aspects of VUR diagnosis and treatment are controversial. We objectively assessed the published data; the data base for many current diagnoses and treatment patterns of VUR is limited. Recent studies have focused on developed determination of VUR-related renal morbidity, improved stratification tools that children would benefit most from which VUR treatment option, and improved reporting of the long-term outcomes of VUR treatments in children who are at risk for VUR. In this review, the advances in the diagnosis and treatment of VUR will be accompanied by the current guidelines.

## 1. Introduction

It has been known for a long time that some urinary tract infections (UTI) can result in acute pyelonephritis (APN) (1). Considering the relationship between vesico-ureteral reflux (VUR) with UTI, APN, renal scarring and end-stage renal failure (ESRF) has been put forth since the middle of the 20th century (2, 3). Continuous antibiotic prophylaxis (CAP) has become a standard treatment in 1970s, by the introduction of safe and effective antibiotics and spontaneous resolution of the reflux (4-6). Ureteroneocystostomy (UNC) has begun to be performed as the surgical treatment in patients who have recurrent and intercurrent UTI, or in whom the VUR did not show spontaneous resolution (7, 8). In the 1980s, the less invasive endoscopic injection (EI) treatment has been used as an alternative therapy of selected patients (9, 10).

As reported in the literatures, in addition to previous renal scars the de novo scar formation was found to be relatively low in VUR patients as a result of two large randomized controlled studies that were evaluating the treatment options (the international reflux trial and the Birmingham reflux trial) (11, 12). In the following studies comparing the medical and surgical treatments of patients with VUR, no difference between the treatment options for prevention of renal injury was observed (13, 14). In recent studies, no differences were found between the low-moderate VUR patients groups who have received and those who have not received CAP; however, CAP was shown to be more effective in high VUR grade (15-17). Some studies still show that VUR alone is not necessary and sufficient for development of renal scarring and acute pyelonephritis, despite the fact that VUR has a clear association with renal scarring (18). As a result of different clinical findings, the failure to make an accurate assessment in diagnosis and treatment of UTI and VUR (19).

## 2. Diagnosis

### 2.1. Diagnosis and Assessment of VUR After Febrile UTI in Children 

The aim of the initial evaluation after a febrile UTI is (1) to prevent recurrent febrile UTI, (2) renal damage, and (3) to minimize morbidity. When the current guidelines for the diagnosis and treatment of VUR are analyzed for this purpose, a detailed anamnesis, physical examination, measurement of the height and weight, blood pressure and serum creatinine level (as it would be a reference for determination of the glomerular filtration rate) seem to be necessary. Urine analysis must be performed for proteinuria and bacteriuria, culture and antibiogram must be performed in the case of infection. In many recent studies, the serum procalcitonin level has been shown to be associated with APN-related renal injury level detected on DMSA scintigraphy and higher erythrocyte sedimentation rate (ESR) and C-reactive protein (CRP). It has been reported that the measurement of the procalcitonin level facilitates the determination of clinically significant VUR and renal injury and decreased the use of VCUG (20-22).

In children, no precise definition characterizing the group of symptoms such as urinary incontinence, dysuria, urinary tract infection, frequency in urine and constipation has been provided (bladder/bowel dysfunction (BBD); dysfunction elimination syndrome (DES), dysfunction lower urinary symptoms); nevertheless, it is important to ask about the very frequent disorders. Bladder/bowel dysfunction is a condition in which storage and excretion diseases such as overactive bladder and urge incontinence, delayed micturation, hypoactive bladder, and micturation dysfunction, may be observed. Although the approach towards children with coexistence of VUR and BBD is not clear, infection-related renal injury has been reported to be high among these children. Untreated BBD may affect the VUR treatment in varying degrees. Among children undergoing CAP, the incidence of concurrent infection has been reported to be higher in those who had BBD compared to those who did not. Resolution of the reflux was observed in 31% of children with BBD and in 61% of children without BBD who have undergone CAP. In VUR patients who had undergone endoscopic surgery, the rate of reflux resolution was determined as 50% in those who had BBD and 89% in children who did not have BBD. It has been reported that the BBD is not affecting the surgical resolution rates which has reported as 97% (23). The EAU (European association of urology) and the ESPU (European society of pediatric urology) guidelines recommend urinary system ultrasonography (USG) and voiding cystourethrography (VCUG) for the initial assessment. The AAP (American academy of pediatrics) recommends only USG in the first assessment and VCUG in the presence of recurrent UTI or ureteral dilation, and renal anomaly on USG. In a prospective study, 88% of post febrile UTI urinary ultrasonographies were normal (24). The EAU, ESPU and AUA (American urological association) guidelines reported that technetium 99m Tc dimercaptosuccinic acid (DMSA) scintigraphy has been recommended in patients who have high grade of VUR, high creatinine levels and intercurrent UTI. As a top-down approach, the ESPR (European society of pediatric radiology) guideline recommends performing USG and DMSA scinitgraphy, to determine the presence of renal dysplasia and acquired renal scar. They recommend performing VCUG in case of renal involvement. This is targeted to reduce the urethral catheterization, the ionizing radiation of the gonads and determination of clinically insignificant VUR. However, the disadvantages of this approach are the high cost and each radiology clinic uses its own isotope (so the results may be different as well as the interpretations) (25). Besides DMSA, the use of 51Cr-ethylenediamineacetic acid has been reported to be effective in the determination of renal function. The use of magnetic resonance urography has also been recommended to determine renal hypotrophy.

### 2.2. Diagnosis and Assessment of Siblings and the Offspring of VUR Patients

VUR is a familial, polygenic, autosomal dominant inherited disease. Although its prevalence is 1% in the general pediatric population, the prevalence is 27% among the siblings of VUR patients and 36% among the offspring of VUR patients. The EAU and the AUA guidelines recommend performing USG initially and VCUG when any abnormalities due to the increased risk are present.

The families should be informed about the VUR risk and families who do not agree to the imaging must be warned about being ready to receive treatment for febrile UTI and the possible necessity of detailed VUR investigation. Although there are no sufficient randomized controlled studies about CAP for the prevention of febrile UTI in siblings and offsprings, the general trend in both guidelines is to prevent renal injury; hence, detection of VUR with screening alone would not be meaningful. A prospective study about this issue still continues in the UK (26).

### 2.3. Diagnosis and Assessment of Prenatal Hydronephrosis

VUR may arise as a prenatal hydronephrosis during the fetal development. Boys are affected more often than girls by VUR arising as antenatal hydronephrosis. Bilateral and high grade VUR are more frequent among boys. However, there is no correlation between the level of prenatal hydronephrosis and the VUR level. The same approach is recommended in prenatal hydronephrosis (screening of siblings and offspring of VUR patients and providing prophylaxis before development of renal injury).

Detection of hydronephrosis or hydroureter on prenatal ultrasonography may support VUR; however, there is no reliable finding for the diagnosis of VUR. Besides post-natal ultrasonography, VUR was determined in 15.2% of children who were found to have mild to moderate prenatal hydronephrosis. Overall, 1/3 of VURs are graded I-II, 1/3 are graded III, and 1/3 are graded IV-V. The SFU (society of fetal urology) and the AUA guidelines recommend VCUG if abnormal bladder findings, hydroureter and SFU grades III-IV prenatal hydronephrosis are detected on fetal ultrasonography (27).

## 3. Treatment

The main purpose of VUR treatment is to protect the patient from febrile UTI, renal injury and accompanying morbidities. Therefore, elements such as age, gender of the patient, the reflux grade, history of recurrent UTI, renal dysfunction and associated bladder-bowel dysfunction must be evaluated and a decision must be made with the family. The treatment approaches are divided into conservative and interventional methods.

### 3.1. Continuous Antibiotic Prophylaxis (CAP)

Skepticisms with regard to the efficacy of CAP, which has become an initial standard therapy for many VUR patients, have begun to increase in recent years. It has been reported that there is no risk for recurrent APG and renal scarring, particularly in children with low grade VUR that most of them have shown spontaneous resolution during the time. In previous studies, CAP has been demonstrated to decrease the risk of febrile UTI until spontaneous resolution. Therefore, in recent studies, compromising of a control group without any utilization of treatment methods or no use of prophylactic drugs was reported to be ethically unfavorable. However, constituting a control group has become acceptable and necessary as the effectiveness of antibiotic prophylaxis has begun to be examined and the infections development due to resistant bacteria have begun to cause problems. Significant studies regarding this issue have been conducted: 

In the PRIVENT (prevention of recurrent urinary tract infection in children with vesicoureteric reflux and normal renal tracts) study carried out in Australia in 2009, a total of 576 children with a mean age of 14 months. In this study low dose of trimethoprim-sulfamethoxazole (TMP-SMX) and placebo were compared. The development of UTI was seen to cause 6% decrease in the antibiotic-receiving group (36/288; 13%) compared to the placebo group (55/288; 19%), and it was concluded that antibiotic prophylaxis had a limited effect (28).

According to the results of a multi-center, placebo-controlled, prospective randomized Swedish reflux trial, as the result of the follow-up of 203 pediatric patients (128 girls and 75 boys) randomly divided into three groups as the placebo, the antibiotic prophylaxis, and the endoscopic injection group, the reflux grade was seen to have decreased in all groups and the VUR had disappeared in 13%, 38% and 15% of the patients in the antibiotic prophylaxis, the endoscopic injection and the follow-up groups, respectively. The improvement rate of the endoscopic injection group (disappearance of reflux and reduction of reflux grade) was found to be significantly higher than the prophylaxis and the follow-up groups. No significant difference was determined between the prophylaxis and the follow-up groups with regards to improvement in the reflux grade. In conclusion, in this randomized controlled study including children with grade 3-4 of VUR, aged between 1-2 years, the antibiotic prophylaxis and endoscopic injection significantly reduced the frequency of febrile UTI in girls compared to the control group. The febrile UTI frequency was found to be three-fold higher in girls of the control group (57%) compared to the antibiotic prophylaxis group (19%). Antibiotic prophylaxis or endoscopic injection could not provide a similar success rate in boys who were above 1 years old and had high grade reflux (29).

The RIVUR (randomized intervention for children with vesicoureteral reflux) study is a multi-center, double blinded, placebo-controlled study that began in May 2007 conducted on 600 children aged between 2 months and 6 years, comparing antibiotic prophylaxis (TMP-SMX) with placebo, and the early results of this study will be published in May 2013. Creation of a more precise algorithm about CAP is targeted as the conservative treatment of VUR (30).

### 3.2. Endoscopic Injection (EI)

This method, which had been defined by Matouschek in 1981, was generalized by Puri and O’Donnell. Teflon (polytetrafluoroethylene) was used as the first subureteric injection substance and various agents were used for the endoscopic injection therapy of VUR. Polydimethylsiloxane, cattle collagen, calcium hydroxylapatite, polyacrylate-polyalcohol co-polymer (Vantris, Promedon, Cordoba, Argentina) and dextranomer/hyaluronic acid (Dx/HA) (Deflux, Oceana Therapeutics, Inc, Edison, NJ, USA) were used. Deflux is the only material in USA, which approved by the FDA (Food and Drug Administration), and it has been generalized since 2001 and become comparable with antibiotic prophylaxis and open surgery techniques. However, the increase in the number of injection treatments with the use of Dx/HA still have not reduced the rate of UNC treatment in some centers.

In the original subureteric Teflon injection method (STING), the bladder mucosa is accessed with the injection needle just below the ureteral orifice located 2-3 mm distal part of the ureterovesical junction and the needle is preceded 4-5 mm into sub mucosal plain. Formation of a crest in the intramural ureter is targeted. This technique was modified by Kirsch and colleagues and the injection needle was enabled to enter the base of the distal ureter through hydrodistention. This modification was developed as the STING procedure could not achieve complete coaptation in the orifice of the ureter. The aim of this technique is to completely close the ureteral tunnel. Researchers have found the success rate of ureteral to about 92% with the hydrodistention-implantation technique (HIT) and 79% with the STING procedure in patients with high grade reflux. Recently, the HIT technique was modified and a better cessation was aimed at the intramural ureter through both distal and proximal injection (double HIT). In this method, the first injection (proximal HIT) is made on the base of the mid-ureteral tunnel and the second injection is made (distal HIT) on the internal of the ureteral orifice. If coaptation cannot be achieved through this method, concurrent STING may be applied additionally. Kirsch et al. achieved a clinical success rate of 96% and a radiological success rate of 96% on the one-year follow-up of 54 patients (31, 32).

The learning curve was found to be related to the VUR rate independently from the injection techniques. Lorenzo et al. showed that surgical experience was an independent predictor for the VUR improvement using the injection treatment (33). The complications of the procedure included contralateral VUR development following unilateral injection treatment, and ureterovesical obstruction, although this was rarely observed. In long terms (mean 22 months), calcification as a result of granulomatous inflammation with pseudocapsule at the injection site may be faulty diagnosed with distal ureteric stone or tumor following the deflux injections.

In a meta-analysis investigating a total of 8101 renal units in a total of 5527 patients who had undergone subureteric injection treatment with all the injection materials (including Dx/HA), the primary success rate was 78.5% in grades I and II of VUR, 72% in grade III, 63% in grade IV and 51% in grade V of VUR. The success rate of the second injection was determined as 68% and the success rate of the third injection was determined as 34% in patients whom the first injection had been failed. The success rate was found to be lower in the duplicated systems than in the single system (50% vs. 73%) and it was lower in patients with neuropathic bladder compared to the patients with normal bladder (62% vs. 74%). 20%-30% of the patients who undergone the injection treatment with Deflux and in VCUG is found to be negative three months after the treatment, present again with recurrent UTI and recurrent VUR (34). Various methods and designs, differences in patient selection, and the follow-up period, the surgical and technical factors have hindered the access to accurate data about effectiveness of EI. Although some studies reported that in the short term (4-6 weeks), the success rate of EI is above 90%, the results of long term follow-up do not support the same results. For example, in the Swedish reflux trial, a 20% recurrence rate was reported as the result of a two-year follow-up of children who had been previously treated with a high success rate (86%). In the same study, the UTI rates were found to be similar in patients who had received prophylaxis and Dx/HA (19% vs. 23%). The rates of infection and renal scarring were found to be similar in the patients who had received Dx/HA and in the patients who had not received any treatment. Some researchers have associated after treatment VUR with the presence of BBD, history of numerous UTI and abnormal DMSA scintigraphy results. The estimations about the cost-effectiveness of EI are variable based on various data. In conclusion, although EI treatment is a beneficial method for patients undergone surgery but further studies are needed for EI to become a permanent treatment method.

### 3.3. Open Ureteroneocystostomy (UNC)

Conventional open UNC is the gold standard in VUR treatment and is associated with low complication rates, high success rates such as 95-98%, and 57% reduction in febrile UTI rates. Rather than the high efficacy, recent studies have focused on the decrease in morbidity by improving the preoperative and postoperative cares, development of postoperative analgesia, reducing the size of the incision and using catheter. Despite various described techniques, the main purpose is to provide the flap valve mechanism to temporarily close the ureter under increased intravesical pressure and also to provide a non-obstructed urinary flow towards the bladder with a normal ureteral peristalsis. The most commonly used method is the cross-trigonal intravesical re-implantation technique described by Cohen. The most important difficulty of this procedure is the difficulty in making the surgical intervention to the urinary stone which may subsequently develop. The other intravesical open surgical procedures are suprahiatal re-implantation (Politano-Leadbetter) and infrahiatal re-implantation (Glenn-Anderson).

Extravesical re-implantation was described by Lich and Gregoir. The most renowned complication of this approach is the risk of increased postoperative temporal urinary retention in patients undergoing bilateral ureteral re-implantation. The success rate has been reported to be the same in extravesical and intravesical ureteral re-implantation (95%) and to be particularly higher in grades I and III of reflux (35, 36).

### 3.4. Laparoscopic/Robot-Assisted Laparoscopic Ureteroneocystostomy (LUNC/RALUNC)

The first LUNC was performed transperitoneally using the extravesical Lich-Gregoir technique. The properties of this technique (post localization, tissue excision margins) were first published by Lakshmanan and Fung. Seventy-one re-implantations were performed in 47 pediatric patients and reflux or obstructions were not observed within the postoperative period. They reported that the ureteral suturing was difficult in children younger than 4 years old and in those with a narrow pelvis, and recommended to change the technique (37). Riquelme et al. reported their success rate as 94.7% in their study on 15 patients with bilateral reflux and duplex systems (38).

Laparoscopic application of the Cohen intravesical re-implantation technique was hindered due to difficulty in installation of the port and limitation of movement, and it was performed through two suprapubic ports and the use of transurethral resectoscope by Gill et al. They reported that this technique was effective and technically proper (39). The common result of the studies has been reported as a decreased need for postoperative analgesia, similar duration of hospital stay and long operative time as an alternative to the open procedure.

RALUNC provides advantages such as reduced pain and morbidity, short learning curve, robotic instruments, high maneuverability, compared with the laparoscopic technique. The RALUNC technique was first begun with the extravesical approach, and the intravesical technique develop due to the increased risk of urinary retention following bilateral extravesical re-implantation. In 2004, Peters performed 17 unilateral extravesical and 3 bilateral intravesical RALUNCs and reported a success rate of 89% and a complication rate of 12% (bladder leakage in 2 patients, temporal obstruction in 1 patient) (40). Casale et al. performed extravesical RALUNC in 41 patients in 2008 and performed the operation by describing and preserving the localized pelvic plexus in the lateral of the ureteral haitus; the success rate was determined as 97.6% and they did not encounter any post-operative complications (including urinary retention). The mean operative time was reported as 2.33 hours. This study eliminated the suspicion about urinary dysfunction developing after bilateral extravesical reimplantation (41). In a recent retrospective study comparing open UNC (39 patients) and RALUNC (39 patients), 22 patients underwent an intravesical operation and 17 patients underwent an extravesical operation in the open surgery group, and 19 patients underwent an intravesical operation and 20 patients underwent an extravesical operation in the robotic surgery group. The operative time was significantly longer in the intravesical and the extravesical RALUNC group. Bladder spasms and hematuria were reported to be less frequent in the intravesical RALUNC group compared to the open intravesical group; however, no significant difference was determined between the groups in terms of pain. The duration of catheter and duration of hospitalization were reported to be shorter in intravesical RALUNC patients. No significant difference was determined between the use of extravesical technique in both groups. The overall clinical and radiological success rates were found to be similar in all groups (42).

## 4. Ongoing Studies and Future Perspectives

It may be considered that the results of the RIVUR study, which will provide more comprehensive data about the effectiveness of continuous antibiotic prophylaxis and the alteration of VUR with regards to the UTI recurrence, renal scar, antimicrobial resistance, quality of life of the patient, compliance to therapy at the end of the two-year follow-up period will provide a change in the diagnosis and treatment of VUR in 2013. Other ongoing experimental studies include warming the intravesical urine non-invasively by using microwave energy and a technique aiming at finding this warm urine in the kidney in order to detect the VUR (43). Another study investigating the role of steroids on the inflammatory response and scarring as the response of the kidney to APN differs in patients, is conducted by NIDDKD (The National Institute of Diabetes and Digestive and Kidney Diseases) (44) (clintrials.gov. study number NCT01391793). The studies aiming at finding more effective agents for prevention of renal infection is increasing.

In conclusion, a few randomized controlled studies and limited number of current studies were analyzed, despite the various options for diagnosis and treatment of VUR, many studies were not superior to one another. Determination of conditions such as age, gender of the patient, degree of VUR, history of UTI, presence of renal scar, and bladder bowel dysfunction will be a guide in choosing the proper treatment method. Independent from the treatment option, the main purpose of VUR treatment is always the same: to protect the patient from ascending UTI and pyelonephritis, to improve micturation, and most important to prevent renal scar formation and renal failure.
